# Paediatric flexible flat foot: how are we measuring it and are we getting it right? A systematic review

**DOI:** 10.1186/s13047-018-0264-3

**Published:** 2018-05-30

**Authors:** Helen A. Banwell, Maisie E. Paris, Shylie Mackintosh, Cylie M. Williams

**Affiliations:** 1 International Centre for Allied Health Evidence, University of South Australia, Adelaide, South Australia 5001 Australia; 2 School of Health Sciences, University of South Australia, Adelaide, South Australia 5001 Australia; 3 Allied Health, Peninsula Health, Frankston, VIC 3199 Australia; 4 School of Primary and Allied Health, Monash University, Frankston, VIC 3199 Australia

**Keywords:** Foot posture, Pes planus, Pes planovalgus, Flat feet, Child, Paediatric, Validity, Reliability, Foot posture index – Six item version (FPI-6), Staheli arch index, Chippaux-Smirak index

## Abstract

**Background:**

Flexible flat foot is a normal observation in typically developing children, however, some children with flat feet present with pain and impaired lower limb function. The challenge for health professionals is to identify when foot posture is outside of expected findings and may warrant intervention. Diagnoses of flexible flat foot is often based on radiographic or clinical measures, yet the validity and reliability of these measures for a paediatric population is not clearly understood. The aim of this systematic review was to investigate how paediatric foot posture is defined and measured within the literature, and if the psychometric properties of these measures support any given diagnoses.

**Methods:**

Electronic databases (MEDLINE, CINAHL, EMBASE, Cochrane, AMED, SportDiscus, PsycINFO, and Web of Science) were systematically searched in January 2017 for empirical studies where participants had diagnosed flexible flat foot and were aged 18 years or younger. Outcomes of interest were the foot posture measures and definitions used. Further articles were sought where cited in relation to the psychometric properties of the measures used.

**Results:**

Of the 1101 unique records identified by the searches, 27 studies met the inclusion criteria involving 20 foot posture measures and 40 definitions of paediatric flexible flat foot. A further 18 citations were sought in relation to the psychometric properties of these measures. Three measures were deemed valid and reliable, the FPI-6 > + 6 for children aged three to 15 years, a Staheli arch index of > 1.07 for children aged three to six and ≥ 1.28 for children six to nine, and a Chippaux-Smirak index of > 62.7% in three to seven year olds, > 59% in six to nine year olds and ≥ 40% for children aged nine to 16 years. No further measures were found to be valid for the paediatric population.

**Conclusion:**

No universally accepted criteria for diagnosing paediatric flat foot was found within existing literature, and psychometric data for foot posture measures and definitions used was limited. The outcomes of this review indicate that the FPI – 6, Staheli arch index or Chippaux-Smirak index should be the preferred method of paediatric foot posture measurement in future research.

**Electronic supplementary material:**

The online version of this article (10.1186/s13047-018-0264-3) contains supplementary material, which is available to authorized users.

## Background

Flexible flat foot (also known as pes planus or planovalgus) in children, when there is the appearance of a lowered medial longitudinal arch, with or without rearfoot eversion [1] is one of the most frequently reported reasons to seek orthopaedic opinion [2]. Yet, in typically developing children, normative data indicates ‘flat’ is normal for children up to eight years of age [3], due to age appropriate osseous and ligamentous laxity, increased adipose tissue and immature neuromuscular control [4, 5]. Although variable, the ‘flatness’ of this foot posture reduces over the first decade of life [3, 6–9]. However, some children with a flexible flat foot posture report lower limb pain [10] and have demonstrated reduced lower limb function [11]. Furthermore, adults with flexible flat feet report significantly increased levels of back and lower limb pain [12] and reduced quality of life [13]. The challenge for health professionals is in identifying when a child’s foot is, or isn’t, in keeping with developmental expectations, particularly in relation to foot posture and/or function; in order to reassure, monitor or intervene accordingly [14, 15]. Therefore, the measure used to indicate where a foot posture is outside of the expected flatness in children (i.e. the diagnoses of flat foot) needs to be valid, reliable and appropriate for developing foot posture typically observed.

Flat foot is diagnosed through a variety of measures, including plain film radiographs (e.g. x-ray), static foot posture measures and footprint analysis [16]. Plain film radiographs are considered the reference standard to determine flat foot magnitude; however, this method is costly, involves radiation risk, and is not routinely used in clinical practice [17]. Plain film radiographs, static postures or footprint methods allow flat foot description by analysing different angles or measures and, in many cases, comparing these to known population norms. The prevalence of paediatric flat foot has been reported as low as 0.6% and as high as 77.9% (age range 5 to 14 years and 11 months to 5 years respectively), [18, 19]. Whilst an explanation of this broad variation may be due to the changing foot posture as the child develops, there is concern that the measures of flat foot may not differentiate between what is an expected level of ‘flatness’ in children and abnormal presentations [3]. To the best of the authors knowledge, there is no comprehensive review of the psychometric properties of flat foot measures as they apply to the paediatric population [16].

The two core elements of psychometric properties are reliability and validity [20]. Reliability relates to the inherent variability of a foot posture measure and the error that is attributable to the rater and the tool used, expressed as the stability of the data when measured by: one observer over two or more occasions (i.e. intra-rater reliability); or two or more observers (inter-rater reliability), [21]. Validity relates to the extent to which a tool measures what it is intended to measure [21]. Validity of a foot posture measure can be expressed in several ways. For example, criterion-related validity would be the ability of one measure of flat foot to predict results of another measure of flat foot that is assumed to be valid, such as comparing a foot print indices to a plain film radiograph as the reference standard [20]. Or construct validity, which in broad terms determines if the measure has enough ‘sensitivity’ to detect when the condition exists (e.g. a measure with high sensitivity has a low level of false-positive diagnoses), and ‘specificity’ to detect when the condition does not exist (e.g. a measure with high specificity has a low level of false-negative diagnoses) [22]. To be confident that a diagnosis of flat foot is correct, the measure used needs to be both valid and reliable for the population to which it’s applied.

The primary aim of this systematic review was to investigate how paediatric foot posture is measured and how paediatric flat foot posture is defined. The secondary aim is to identify the psychometric properties of the foot posture measures used to determine if these measures are valid and reliable for this population.

## Methodology

### Protocol and registration

The systematic review was guided by the PRISMA protocol [23]. The registered protocol is listed on PROSPERO, registration number: CRD42016033237.

### Information sources and search strategy

The following databases were searched from inception to Jan 2017: MEDLINE [Ovid], CINAHL, EMBASE, The Cochrane Library, AMED, SportDiscus, PsycINFO, and Web of Science. The search terms are outlined within Table 1.

**Table 1:** Search terms for systematic review of the literature on flexible flat foot in paediatrics

Search Terms	
Foot/ OR Feet	
AND	
Child/ OR Infant/ OR asolescen*/ OR “preschool”/	
AND	
posture*/ OR “biomech*”/ OR “footprint*”/ OR “morphology*”/ OR “navicular height”/ OR “foot posture ind*”/ OR “p?ediatric flat foot proforma”/ OR “arch ind*”/ OR “arch height ind*”/ OR “foot mobility magnitude”/ OR “hindfoot posture”/ OR “arch insert”/ OR “medial arch”/ OR “foot posture measure*”/ OR “foot function ind*”/ OR “p-ffp” [paediatric flat foot proforma]/ OR “pffp” [paediatric flat foot proforma]/ OR “fpi”/ OR “fmm”/	

**Fig. 1:**
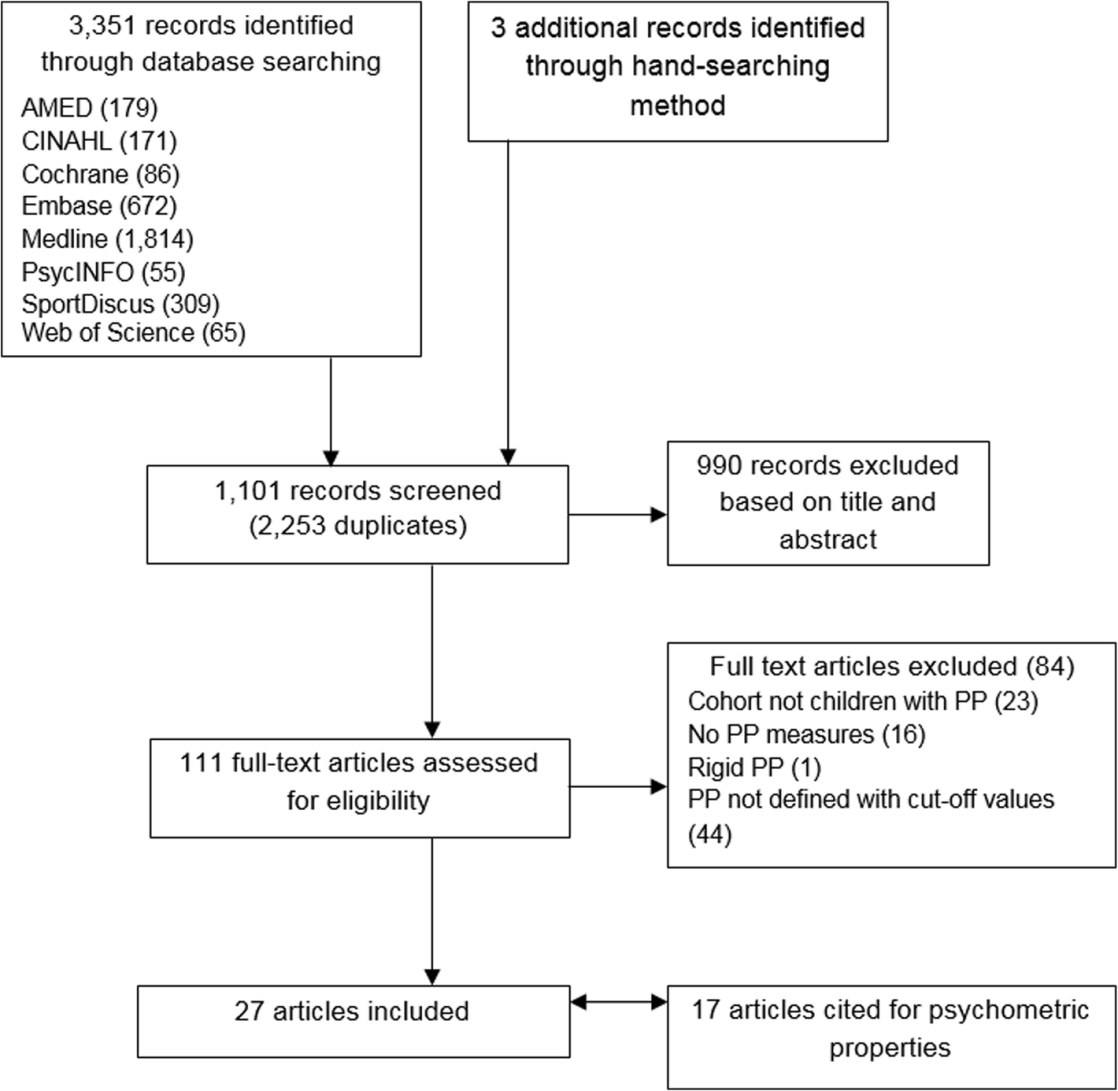
Flow chart of search strategy

Medical subject headings (MeSH) were exploded, combined with relevant keywords and truncated as necessary. Searches were limited to English language studies. Further studies were sought from a review of reference lists, conference proceedings and personal communications with content experts (Fig. 1). In addition, studies referenced within the final included articles that cited psychometric properties of the measures and criteria used to define flat foot were sourced (Fig. 1).

### Eligibility criteria

Studies were included if published in peer-reviewed journals, participants were aged ≤18 years and the outcomes included a definition and measure of flat foot. Table 2 displays the full inclusion and exclusion criteria.

**Table 2:** Inclusion and exclusion criteria

Inclusion	Exclusion
Sample included individuals with pes planus	Participants with a history of rigid pes planus
Definition of pes planus, with criteria described	> 18 years of age
Conducted/described measures, which were aimed at diagnosing pes planus (e.g. rearfoot posture, arch height and footprint measures)	Participants who had acutely painful or inflammatory conditions (e.g. juvenile arthritis)
Children (≤18 years of age)	
Empirical studies	
English language	

Title, abstract and full-text screening was independently conducted by two investigators (MP, HB/SM) with a third reviewer (CW) consulted in the event of non-agreement (Fig. 1).

#### Critical appraisal of bias and data extraction

A priori decision was set to include all studies meeting the criteria regardless of potential risk of bias and include all measures of flat foot where validity and reliability measures reached a moderate or above rating (see data management for rating parametres), [22, 24, 25]. Data extraction was in keeping with the aims of the study and included; study design, participant age range, sample size, ethnicity/country of study, foot posture measure(s), flat foot definition and relevant psychometric data related to QAREL and a purpose-built criterion described below.

The outcomes of interest in validity studies were sensitivity, specificity and correlation with a reference standard (e.g. plain film radiographs). Validity was assessed with a purpose-built criterion (Additional file 1), covering: reported validity of the flat foot measure and definition; age (in years) of the test population; differences in the cited protocol reported and included study protocol; and, a pragmatic determination of whether validity was demonstrated for a paediatric population (yes/no/with caution). For example, a yes was assigned if a paediatric sample was used for validity testing, the study protocol matched the cited protocol and sensitivity / specificity or correlations with reference standard were moderate or above; a no would be assigned if the study population was adult or sensitivity/specificity or correlations with reference standard were below moderate. With caution was assigned if the study population had been paediatric but aspects of sensitivity/specificity or correlation with a reference standard had mixed results (Additional file 1).

The reliability outcome of interest was inter-rater agreement. Inter-rater reliability studies were appraised using the QAREL checklist [26, 27] and a purpose-built assessment (Additional file 1). The 11 item QAREL tool assesses: if the test evaluated a sample of representative subjects; was it performed by raters representative of those standardly using the measure; were raters blinded to i) the findings of other raters, ii) their own prior findings iii) the reference standard outcomes, iv) other clinical information, and v) cues that were not part of the procedure; was the order of examination randomised; was the time interval between measures suitable; did they apply the protocol appropriately; and, was the statistical analysis correctly conducted. Each item was scored as yes, no, unclear or not applicable rating. The QAREL score is the number of items that received a ‘yes’ rating (Additional file 1). The purpose-built criteria covered five criteria; definition of flat foot used, age (in years) of the test population; differences in protocol reported between the cited and included article; inter-rater reliability measure and outcome; and, a pragmatic determinant of whether reliability was demonstrated for a paediatric population (yes/no/with caution). The assignment of yes/no/with caution were based on similar outcomes as for validity ratings (Additional file 1).

Two investigators independently extracted data and assessed articles against the QAREL criteria and purpose-built criteria (HB, MP/CW) with any discrepancies resolved by a fourth reviewer (SM).

#### Data management

**Fig. 2:**
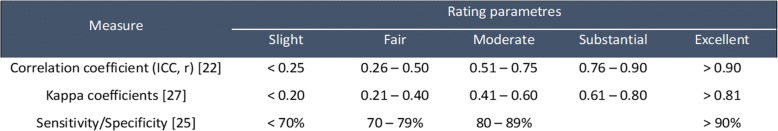
Rating parametres applied

Data were synthesized in table form. Correlations with reference standards and inter-rater reliability outcomes were presented as Intraclass Correlation Coefficients (ICCs) [22], kappa coefficients [27] or sensitivity and specificity data [25]. For consistency, outcomes were rated according to Fig. 2. All other responses displayed as descriptive only or awarded a yes/no/with caution response. Outcomes were required to be deemed valid and reliable to be accepted as appropriate.

Due to the heterogeneity of the included studies, a meta-analysis was not conducted. Instead, a descriptive synthesis of the results was undertaken.

## Results

### Study selection

The search strategy identified 1101 unique titles (Fig. 1). Following screening, a total of 27 articles were included in the review.

### Participants

A total of 15,301 child participants were included within the 27 studies (Table 3). Participants ranged between 3 and 18 years of age. Sample sizes ranged from 22 to 5866 (Table 3). In one study, all participants were male [28]. Four studies separated participants into overweight and normal weight groups for analysis [29–32]. Ethnicity or country of study was reported in 26 studies, representing 15 different ethnicities or countries (Table 3).

**Table 3:** Summary of included studies

Author (date)	Study code	*N*	Study design	Study aim	Participants	Mean age (SD), range in years*	Ethnicity or Country of study	Foot posture measure used
Abolarin et al. (2011)	[45]	560	Cross-sectional	To determine the role of age and type of foot wear as predictors of flatfoot	School children	6–12	Nigerian	Instep
Aharonson, Arcan & Steinback (1992)	[53]	82	Case-series	To establish foot-ground pressure patterns	Children with flexible flat foot	4–6	Caucasian	Rearfoot eversion
								Foot ground pressure
								Plantarflexion of talus angle
								Calcaneal pitch angle
								AP talocalcaneal angle
Bok et al. (2016)	[33]	21	Cohort	To evaluate the effects of different foot orthoses inversion angles on plantar pressure during gait	Children with flexible flat foot	9.9 (1.6), 8–13	South Korean	Rearfoot eversion (plus one of the following)
								AP talocalcaneal angle
								Lateral talocalcaneal angle
								Talus-first metatarsal angle
								Calcaneal pitch angle
Chang et al. (2014)	[46]	1228	Cohort	To establish a new classification of flatfoot by characteristics of frequency distribution in footprint indices	School children	7.3 (1.1), 6–10	Taiwanese	Staheli arch index
								Chippaux-Smirak index
Chen et al. (2011)	[34]	1319	Cohort	To analyse and compare footprint measures of preschool aged children	Children with flexible flat foot	5.2, 3–6	Taiwan	Clarke’s angle
								Chippaux-Smirak Index
								Staheli arch index
Chen et al. (2014)	[56]	605	Cohort	To determine the prevalence of flatfoot in children with delayed motor development	Children with & without developmental coordination disorder	4.4, 3–7	Taiwanese	Chippaux-Smirak index
Chen et al. (2015)	[54]	21	Cohort	To investigate the effects of foot wear on joint range of motion, ground reaction forces and muscle activity	Children with & without flat foot	6.3, 5–11	Taiwanese	Arch index
Drefus et al. (2017)	[47]	30	Cross-sectional	To determine the intra and inter-rater reliability of the Arch height index	Children	9.6 (2.0), 6–12	United States	Rearfoot eversion
								Arch height index (sitting/standing)
Evans and Karimi (2015)	[29]	728	Cross-sectional	To explore the relationship between foot posture and body mass	Over and normal weight children	9.1 (2.4), 3–15	*Australia and United Kingdom*	FPI-6
Ezema et al. (2014)	[48]	474	Cross-sectional	To determine associated personal characteristics of flatfooted school children	Children	6–10	*Nigerian*	Staheli arch index
Galli et al. (2014)	[35]	70	Cohort	To determine if children with Down syndrome were characterised by an accentuated external foot rotation in gait	Children with & without Down syndrome	9.6 (1.7), 4–14	Italy	Arch index
Galli et al. (2015)	[36]	64	Cohort	To characterise quantitatively the foot-ground contact parameters during static upright standing	Children with & without cerebral palsy	8.6 (2.4), 5–13	Italy	Arch index
García-Rodríguez et al. (1999)	[49]	1181	Cross-sectional	To estimate prevalence and number of unnecessary treatments of flatfooted children	School children	4–13	*Spanish*	Plantar footprint
Kothari et al. (2016)	[50]	95	Cross-sectional	To investigate the relationship between foot posture and the proximal joints	Children with & without flat foot	11 (2.9), 8–15	United Kingdom	Arch height index
Morrison, Ferrari & Smillie (2013)	[28]	22	Quasi-RCT	To report clinical findings of foot posture and lower limb hypermobility and evaluate the impact of foot orthoses on spatio-temporal gait parameters.	Male children with developmental coordination disorder	Median age 7.5, 6–11	United Kingdom	FPI-6
Nikolaidou & Boudolos (2006)	[37]	132	Cohort	To develop a footprint-based classification technique for the rational classification of foot types	School children	10.4 (0.9), 9–11	Greek	Arch index
								Chippaux-Smirak index
								Martirosov’s K index
								Clarke’s angle
Pau et al. (2016)	[30]	130	Cohort	To screen plantar pressures during level walking with a backpack among normal, overweight and obese school children	Overweight, obese and normal weight children	9.3 (2.0), 6–13	Italian	Arch index
Pauk, Ihnatouski & Najafi (2014)	[38]	93	Cohort	To assess differences in plantar pressure distributions and reliability of the Clarke’s angle	Children with & without flat foot	12.6 (1.9), 9–16	Poland	Clarke’s angle
								Calcaneal pitch
								Calcaneal first metatarsal angle
Pauk & Szymul (2014)	[55]	73	Case-control	Comparing vertical ground reaction force data between flat and neutrally aligned feet	Children with & without flat foot	10.8 (3.6), 4–18	Poland	Clarke’s angle
								Rearfoot eversion
Pfeiffer et al. (2006)	[39]	835	Cohort	To establish prevalence and cofactors of flatfoot, and estimate number of unnecessary interventions received	Children	3–6	Austrian	Rearfoot eversion
Reimers, Pedersen & Brodersen (1995)	[40]	759	Cohort	To establish foot deformity and triceps surae length in Danish children	Children and adolescents	3–17	Denmark	Chippaux-Smirak index
Selby-Silverstein, Hillstrom & Palisano (2001)	[41]	26	Cohort	To determine if foot orthoses immediately affected gait of children with Down syndrome or excessively pronated feet	Children with flat foot, with & without Down syndrome	3–6	North American	Rearfoot eversion
Stavlas et al. (2005)	[51]	5866	Cross-sectional	To determine foot morphology evolution in children between 6 and 17 years of age	Children	6–17	Greek	Footprint evaluation
Tashiro et al. (2015)	[52]	619	Cross-sectional	To investigate the relationship between toe grip strength and foot posture	Children	11.2 (0.7), 10–12	Japan	Staheli arch index
Twomey et al. (2010)	[42]	52	Cohort	To investigate differences in kinematics during walking gait	Children with & without flat foot	11.2 (1.2), 9–12	Not reported	Clarke’s angle
								Arch index
								Navicular height
Villarroya et al. (2009)	[31]	116	Case-control	To evaluate the measures of, and foot arch types, in different weight children using radiographic and footprint indices	Obese & non-obese children	Boys 12.4 (1.6), Girls 11.9 (1.5), 9–16.5	Spanish	Clarke’s angle
								Chippaux-Smirak index
								Calcaneal pitch
								Talus-first metatarsal angle
Yan et al. (2013)	[32]	100	Case-control	To examine changes in dynamic plantar pressure distribution in children of different weight	Obese & non-obese children	10.3 (0.7), 7–12	China	Arch index

*where available

AP – anteroposterior, FPI-6 – foot posture index – 6 item, LAC - longitudinal axis of calcaneus, LAF - longitudinal axis of foot, MLA – medial longitudinal arch, NR – not reported, mm – millimetres

Additional information regarding foot posture parametres can be found in Additional file 2

### Study design

The majority of included studies were cohort [30, 33–44] and cross-sectional [29, 45–52], with a respective 13 and 9 of each study design. Of the other five included articles, three were case control [31, 32, 38], one was a case series [53], and one was a quasi-randomised controlled trial [28].

### Primary findings

#### Foot posture measures and definitions

Across the 27 included studies, 20 foot posture measures were used, involving 40 definitions of flat foot (Table 4). Ten of the 27 studies used multiple measures of flat foot. One study featured a novel method of footprint evaluation [51]. Methodological variations existed across studies, with different parameters and angles assessed following measurement, and different methods for obtaining the footprint/angle and determining flat foot (Table 4, Additional file 2).

**Table 4:** Rating of reported validity and reliability for foot posture measures and definition of flexible flat foot in paediatric populations

Foot posture measure		Study code	Flat foot definition used	Age range of participants in years	Validity as reported in paediatric population	Reliability as reported in paediatric population	Rating of validity/reliability (Yes/No/With caution)
Plain film radiograph angles	Calcaneal pitch	[33, 53]	< 20°	4–6 & 8–13	Nil	Nil	No/No
		[38]	< 23°	4–18	Nil	Nil	No/No
		[31]	≤ 15.4°	7–12	NR [57]	Nil	No/No
	AP talocalcaneal	[53]	> 25°	4–6	Nil	Nil	No/No
		[33]	> 30°	8–13	Nil	Nil	No/No
	Plantarflexion of talus	[53]	> 23°	4–6	Nil	Nil	No/No
	Lateral talocalcaneal	[33]	> 45°	8–13	Nil	Nil	No/No
	Calcaneal first metatarsal	[38]	145°-170°	4–18	Nil	Nil	No/No
	Talus-first metatarsal	[33] [31]	> 4°	7–13	Nil	NR [80], NA [64]	No/No
Foot print indices	Arch index	[35, 54], [36]	≥ 0.26	3–6, 5–13, 4–14	Nil	Nil	No/No
		[37]	≥ 0.26	10	NR [58]	Substantial [81], NR [37]	No/Yes
		[30, 32, 42]	> 0.26	6–13	Nil	Nil	No/No
	Chippaux-Smirak	[46]	≥ 59%	6–9	Nil	Excellent [46]	No/Yes
		[34]	> 62.7%	3–7	Moderate [34]	NR [65]	With caution/No
		[56]	> 62.7%	3–7	Moderate [34]	Nil	With caution/No
		[37],	≥ 45%	10	NR [59]	NR [37]	No/No
		[40]	≥ 45%	3–17	Nil	Nil	No/No
		[31]	≥ 40%	9–16	Moderate [31] NR [60]	Nil	With caution/No
	Clarke’s angle	[34]	≤ 14.04	3–6	Moderate [34]	Nil	With caution/No
		[37]	≤ 20°	10	Nil	NR [37, 59]	No/No
		[38]	< 42°	9–16	Excellent [38]	Nil	With caution/No
		[55]	< 42°	4–18	Nil	Nil	No/No
		[31]	< 29.9°	9–16	Moderate [31],NR [60]	Nil	With caution/No
	Staheli arch index	[46]	≥ 1.28	6–9	Nil	Excellent [46]	No/Yes
		[34]	> 1.07	3–6	Moderate [34]	NR [65]	With caution/No
		[48]	> 1.15	6–10	NR [59, 61]	Nil	No/No
		[52]	> 0.89	10–12	Nil	Nil	No/No
	Footprint index	[42]	< 0.25	9–12	Nil	Nil	No/No
	Martirosov’s K index	[37]	≥ 1.25	10	Nil	NR [37]	No/No
	Footprint evaluation	[51]	X > Y	6–17	Nil	NR [66]	No/No
	Instep	[45]	100 mm	6–12	Nil	Nil	No/No
	Plantar footprint	[49]	≥ 50%	4–13	Nil	Nil	No/No
Static foot measures	Rearfoot eversion	[53]	> 10°	4–6	Nil	Nil	No/No
		[33]	≥ 4°	8–13	Nil	Nil	No/No
		[47]	≥ 4°	6–13	Nil	NA [67]	No/No
		[55]	> 5°	4–18	Nil	Nil	No/No
		[39]	> 5°	3–6	Nil	NR [68]	No/No
		[41]	> (7° - age)	3–6	Nil	Substantial [41]	No/Yes
	Arch Height Index	[47]	≤ 0.37	6–13	NR [62]	Substantial [47]NR [82, 83]	No/Yes
		[50]	< 0.31	8–15	Nil	Nil	No/No
	FPI-6	[29]	≥ + 6	3–15	Not rated^ [63]	Substantial [69]	With caution/Yes
		[28]	≥ + 4	6–11	Nil	Excellent [70]	No/Yes
	Navicular height	[42]	< 20 mm	9–12	Nil	Nil	No/No
Other measures	Plantar pressure analysis (FGP)	[53]	54%	4–6	Nil	Nil	No/No

AP – anterioposterior, FPI-6 – foot posture index – 6 item version, NR – not reported in cited text, NA – not available, FGP – foot ground pressure

Notes: See data management for ratings of reliability and validity. *See Additional file 1 for rating parametres. ^RASCH analysis

Of the 20 foot posture measures used, six were plain film radiographs of angles including calcaneal pitch (or calcaneal inclination), anterior-posterior talocalcaneal (AP talocalcaneal), plantarflexion of talus, lateral talocalcaneal, calcaneal-first metatarsal and talus-first metatarsal angles (Table 4). Nine were footprint indices (Chippaux-Smirak index, Arch index, Clarke’s angle [or Footprint angle, Alpha angle], Staheli Arch index, Footprint index, Martirosov’s K index, Footprint evaluation, Instep and Plantar footprint), (Table 4). There were four static foot measures (rearfoot eversion, Arch height index, Foot Posture Index–6 item version [FPI-6] and navicular height) and one plantar pressure study [Foot Ground Pressure], (Table 4).

The Arch index was the most frequently used measure (*n* = 7), with the Chippaux-Smirak index and rearfoot eversion also frequently employed (*n* = 6 respectively), (Table 4). A further seven measures were used in more than one study (Clarke’s angle (*n* = 5), Calcaneal pitch and Staheli arch Index (*n* = 4), and, AP talocalcaneal, Talus-first metatarsal angle, Arch height index and FPI-6 (*n* = 2 respectively)), (Table 4). Nine alternate assessment measures were used once across the included studies: plantarflexion of talus, lateral talocalcaneal angle, calcaneal-first metatarsal angle, and; Footprint index; Martirosov’s K Index; instep; Plantar Footprint; navicular height; and, Foot Ground Pressure (Table 4).

The most commonly used flat foot definition was the Arch Index ≥0.26, used four times across the 27 included studies. An Arch index of >0.26 was used twice, and ≥0.28 used once in three further studies. A Chippaux-Smirak Index of ≥45 and >62.70% were used twice (n = 2 respectively). Other definitions used twice across the included studies were talus-first metatarsal angle, rearfoot eversion 5° and 4°, and a Clarke’s Angle of <42° (Table 4).

Thirteen of the included 27 studies did not investigate or report the psychometric properties of the measures used to determine paediatric flat foot [30, 32, 33, 35, 36, 40, 45, 49, 50, 52–55], (Table 4), leaving 8 of the 20 foot posture measures used within this systematic review without reported validity or reliability outcomes to justify their use. Specifically; plain film radiograph measures of AP talocalcaneal angle, plantarflexion of talus, lateral talocalcaneal angle, calcaneal first metatarsal angle; the Instep; Plantar footprint; navicular height; and, Foot Ground Pressure, (Table 4, Additional file 1).

#### Quality and appropriateness of reported psychometric properties for a paediatric population

Two studies investigated the validity of the foot posture measures used with their studies [34, 38], five studies [29, 37, 47, 48, 56] justified their choice by citing seven existing studies [57–63] and one study did both [31]. No foot posture measures were assessed with a ‘yes’ ranking in relation to their validity for a paediatric population (Table 4, Additional file 1). The Chippaux-Smirak index, Clarke’s angle, Staheli arch index and the FPI-6 respectively were ranked as relevant to a paediatric population ‘with caution’ (Table 4, Additional file 1).

The quality of the reliability testing, in relation to a paediatric population, was also limited. Four studies investigated the reliability of the measure used to determine flat foot within their studies [37, 41, 46, 47], five studies [28, 29, 34, 39, 51] justified their choice by citing seven existing studies [64–70] and three studies did both [31, 37, 47]. Two cited articles were not available to assess [64, 67]. The Arch index, Chippaux-Smirak index, Staheli arch index, rearfoot eversion, Arch height index and the FPI-6 received a ‘yes’ ranking as relevant to their reliability for a paediatric population (Table 4, Additional file 1), with only the Chippaux-Smirak index, the Staheli arch index and rearfoot eversion reported as having almost perfect repeatability within this population (Table 4). However, alternative studies investigating the Chippaux-Smirak index, Staheli arch index and the FPI-6, as well as the Clarke’s angle were assessed as relevant to a paediatric population ‘with caution’ (Table 4, Additional file 1).

### Summary of results

From the 27 studies included, data were extracted for 20 foot posture measures involving 40 definitions of flat foot within a paediatric population (Table 3). Eight of the included 27 articles investigated the reliability or validity of the flat foot measures used, six further articles justified their choice of measure by citing existing psychometric data and 13 articles neither justified nor reported psychometric properties for their measures of choice (Table 4). Seven measures, involving 11 definitions of flat foot, were determined to have reported validity or reliability specific for a paediatric population (Table 4). Of these measures, no measure had strong data to support validity and reliability of the measure in paediatric samples, and only three were reported to have moderate or with caution validity data and moderate or above reliability data for a paediatric population. Specifically, these three measures were the Chippaux-Smirak index of >63%, ≥59% and ≥40% (for children aged six to nine, three to seven and nine to 16 years respectively), the Staheli arch index of >1.07 and ≥1.28 (for children aged three to six and six to nine respectively) and the FPI-6 of ≥ + 6 (for children aged three to 15 years), (Table 4).

## Discussion

There was a modest body of evidence reporting paediatric specific measures of foot posture. There was no consistently used measure to determine paediatric flexible flat foot in the literature and the choice of foot posture measure, in relation to the validity and reliability, was rarely justified. Within the scope of this review, only three measures of flexible flat foot had any published data to support validity and reliability of the measure within a paediatric population; the Chippaux-Smirak index, Staheli arch index and the FPI-6. However, each of these measures were deemed to have limitations.

The Staheli arch and Chippaux-Smirak, used four and six times respectively across this review, are foot print indices, based on the width of the midfoot compared to the width of the rearfoot (Staheli arch) or metatarsals (Chippaux-Smirak), when the foot is in bipedal weight-bearing relaxed stance, expressed as a ratio (Additional file 2). As the child’s arch develops with age, the ratio should decrease accordingly. This is supported by normative data [3]. The definition of flat foot for the Chippaux-Smirak index within this review did decreased linear to age: 62.7% in 3 to 6 year olds, to ≥40% in 9 to 16 year olds (Table 3). However, the definitions of flat foot for the Staheli arch index did not decrease as expected (e.g. >1.07 in 3 to 6 year olds and ≥1.28 in 6 to 9 year olds, Table 3). This finding is not consistent with existing normative data and suggests these definitions should be used with caution. Furthermore, concerns exist that two-dimensional indices are limited in their ability to assess a three-dimensional construct [71]. It is suggested that categorising the foot posture based on footprint data disregards the complexity and multi-planar motion of the foot [3]. This greatly challenges the validity of the measures using this construct. At a minimum, these measures are reportedly influenced by the weight of the participants [72].

The FPI-6 is a composite tool that assesses multiple components of foot posture, relative to the age of the participant, and presents as an overall score between − 12 to + 12 [73], (Additional file 2). The ‘with caution’ rating assigned to the validity of the FPI-6 was due to the results including an adult population [63]. A flat foot definition ≥ + 6 for a paediatric population is well supported in the literature in terms of normative data [3, 69, 74, 75] and it is considered as the only flat foot scale that accommodates differences between normal and overweight/obese children [29]. Furthermore, only the FPI-6 was tested with a broad age range (i.e. children aged 5 to 16 years old [69, 70]). Interestingly, the FPI-6 was only used in two of the include studies [28, 29], despite being the recommended foot posture measure associated with the GALLOP proforma [76] (an opinion and evidence based proforma for assessment of gait and lower limbs in paediatrics).

The topic of paediatric foot posture remains controversial [39, 77] with little consensus on how this frequently observed foot type should be measured, defined or assessed. Importantly, it is acknowledged that a flat foot posture outside of expected norms may not require management. Clinician’s evaluation of the child, directed by a validated tool such as the paediatric flat foot proforma (p-FFP) [15] assist the clinician in determining when intervention may be required. What this review has highlighted, however, is an issue central to the discourse surrounding this topic. That is, much of the evidence that guides clinician assessment and intervention into paediatric flexible flat foot are potentially based on unsubstantiated measures. It is essential this is addressed in future research. Valid and reliable diagnoses of flat foot appropriate to the paediatric population is required to i) inform the clinician when the foot posture is not in keeping with expected development, and ii) allow research to be appropriate and clinically applicable.

Considering the difficulties associated with static foot print analysis, researchers and clinicians may need to consider the FPI-6 or alternative composite tools (such as the foot mobility magnitude model [78]) or dynamic measurement to better understand paediatric foot structure. Indeed, paediatric based studies have shown a significant difference between static structure and dynamic foot function [79] which may be of clinical relevance. As there was a paucity of dynamic measures in the included studies, further investigation may be beneficial. This extends also to a lack of understanding on the ability of these measures to detect change over time. For researchers to adequately assess development of, and intervention effects in, paediatric flexible flat foot, measures need to be robust and applicable.

There are a number of key limitations in this study. Only English language studies were included in the search strategy and the risk of bias of the included studies was not assessed with a specific critical appraisal tool. Many of the included studies did not cite support for their choice of measure or did not cite appropriately. Indeed, many of the studies reporting existing data assumed it was obtained appropriately and transferrable to their study. For example, Villarroya et al. (2009) quoted psychometric data for the Chippaux-Smirak index from the Kanatli, Yetkin and Cila (2001) article, which relates to the validity of the Staheli arch index; and Mathieson et al. (1999) was quoted in Nikolaidou et al. (2006) even though it obtained data from an adult population. Many studies did not describe their methods or population clearly (Table 2), and two texts were unavailable to the authors [64, 67]. Therefore, these results should be interpreted accordingly. This systematic review was also limited by a paucity of literature in relation to foot posture assessment in the paediatric population. Within the limits of this study, even the reference standard measures (e.g. plain film radiographs) had little psychometric data. Although this review had a broad scope, it did not account for studies which looked solely at the psychometric properties of a measure without a definition of pes planus. Therefore, future studies may search for these measures individually. Furthermore, this systematic review process was underpinned by best practice in the conduct of systematic reviews (PRISMA), however, potential publication and language bias should be acknowledged.

## Conclusion

A synthesis of available literature reveals that there is not a universally accepted criterion for diagnosing abnormal paediatric flat foot within existing literature, and psychometric data for the measures and definitions used was limited. Within the limits of this review, only three measures of flexible flat foot had any published data to support validity and reliability of the measure within a paediatric population (Chippaux-Smirak index, Staheli arch index and FPI-6), each with their own limitations. Further research into valid and reliable, clinically relevant foot posture measures, including dynamic measures and the influence of age, gender and body mass on flat foot incidence, specifically for the paediatric population, is required. Furthermore, age-specific cut-off values should be further defined.

## Additional files

Additional file 1: **Table A1.** Reported validity data and population observed from included studies. **Table A2.** Reported validity data, population and protocol observed from cited studies. **Table A3.** Reported inter-rater reliability, population observed and QAREL score for included data. **Table A4.** Reported inter-rater reliability, population observed, protocol observed and QAREL score for cited data. **Table A5.** QAREL checklist outcomes for inter-rater reliability data of included and cited articles. (DOCX 35 kb)

Additional file 2: **Table A4.** Summary of foot posture tools. (DOCX 4276 kb)

## Abbreviations

AP: anteroposterior
FGP: foot ground pressure
FPI-6: foot posture index – 6 item
GALLOP: Gait and Lower Limb observations of Paediatrics proforma
ICCs: Intraclass Correlation Coefficient
LAC: longitudinal axis of calcaneus
LAF: longitudinal axis of foot
MLA: medial longitudinal arch
NA: not available
NR: not reported
