# Supplementary material for: Paediatric flexible flat foot: how are we measuring it and are we getting it right? A systematic review
Source: J Foot Ankle Res. 2018 May 30;11:21. doi: 10.1186/s13047-018-0264-3 (PMC5975578; doi:10.1186/s13047-018-0264-3)
Supplement: Supplementary file 1 — Table A1. Reported validity data and population observed from included studies. Table A2. Reported validity data, population and protocol observed from cited studies. Table A3. Reported inter-rater reliability, population observed and QAREL score for included data. Table A4. Reported inter-rater reliability, population observed, protocol observed and QAREL score for cited data. Table A5. QAREL checklist outcomes for inter-rater reliability data of included and cited articles. (DOCX 35 kb) [file 13047_2018_264_MOESM1_ESM.docx]

**Additional file 1**

**Table A1:** Reported validity data and population observed from included studies.

| **Measure** | **Study code** | **Definition of flat foot used** | **Age of population tested (Mean age (SD), range in years*)** | **Sensitivity** | **Specificity** | **Correlation to plain film radiograph (angle)** | **Other measure of validity** | **Was validity reported within a paediatric population (yes/no/with caution)** | **Cautions/ Reasons for exclusion** |
| --- | --- | --- | --- | --- | --- | --- | --- | --- | --- |
| Arch index | [43] | ≥0.26 | 10.4 (0.9), range 9 - 11 | Not tested | Not tested | Not tested | Co-classification with KI, FPA and CSI (all < 30%) | No | Validity not established |
| Chippaux-Smirak Index | [32] | >62.7% | Median age 5.2, range 3 - 6 | 0.87 | 0.88 | Not tested | Nil | With caution | Sensitivity and specificity tested against clinical (observational) diagnoses of pes planus |
|  | [53] | ≥40% | Boys 12.4 (1.6), Girls 11.9 (1.5), range 9 -16.5 | Not tested | Not tested | *r* = 0.51 (talus-first metatarsal)  *r* = 0.51 (calcaneal pitch) | Nil | With caution | Participants are potentially outside of ‘developing’ foot age and only moderate correlation with plain film demonstrated |
| Clarke’s angle | [32] | 14.04 degrees | Median age 5.2, range 3 - 6 | 0.86 | 0.88 | Not tested | Nil | With caution | Sensitivity and specificity tested against clinical (observational) diagnoses of pes planus |
|  | [43] | ≤29.9 degrees | 10.4 (0.9), range 9 - 11 | Not tested | Not tested | Not tested | Co-classification with AI, KI, FPA and CSI (all < 30%) | No | Validity not established |
|  | [45] | <42 degrees | 12.6 (1.9), range 9 - 16 | Not tested | Not tested | *r* = 0.9 (calcaneal pitch &  calcaneal–first metatarsal) | Nil | With caution | Participants potentially outside of ‘developing’ foot posture age |
|  | [53] | <29.9 degrees | Boys 12.4 (1.6), Girls 11.9 (1.5), range 9 -16.5 | Not tested | Not tested | *r* = 0.51 (talus first metatarsal)  *r* = 0.51 (calcaneal pitch) | Nil | With caution | Participants potentially outside of ‘developing’ foot posture age and only moderate correlation with plain film demonstrated |
| Staheli arch index | [32] | > 1.07 | Median age 5.2, range 3 - 6 | 0.89 | 0.81 | Not tested | Nil | With caution | Sensitivity and specificity tested against clinical (observational) diagnoses of pes planus |
| Martirosov’s K | [43] | ≥1.17 | 10.4 (0.9), range 9 - 11 | Not tested | Not tested | Not tested | Co-classification with AI, KI, FPA and CSI (all < 25%) | No | Validity not tested |

***** where reported

Study codes:

**31 - Chang et al. 2014; 32 - Chen et al. 2011; 35 – Drefus et al. 2017; 43 - Nikolaidou & Boudolos 2006; 45 - Pauk, Ihnatouski & Najafi 2014; 49 - Selby-Silversterin; 53 – Villarroya et al. 2008**

**Table A2:** Reported validity data, population and protocol observed from cited studies.

| **Measure** | **Study ref** | **Definition of flat foot used** | **Age of population tested (Mean age ± (SD), range in years)** | **Protocol applied** | **Sensitivity reported** | **Specificity reported** | **Correlation to plain film radiograph (angle)** | **Other measure of validity** | **Was validity reported within a paediatric population (yes/no/with caution)** | **Cautions/ Reasons for exclusion** |
| --- | --- | --- | --- | --- | --- | --- | --- | --- | --- | --- |
| Calcaneal pitch | [57]  Text unavailable | ≤ 15.4° | Abstract reported ‘adult’ | Unknown | Unknown | Unknown | Unknown | Nil | No | Adult population |
| Arch index | [60] | ≥0.26 | Mean age 63.3 (13.1) | Footprint divided into thirds (excluding toes) by parallel lines that are perpendicular to the foot axis, identified by the distal and proximal points. Arch index is ratio of midfoot area to area of the entire foot | Not tested | Not tested | Not tested | r=0.67 to navicular height | No | Elderly population |
| Chippaux-Smirak Index | [64] | NR | 6.4, range 3.7 – 11.7 | A static footprint was recorded for each foot during half body weight–bearing position. For each foot, the widest part of the arch and the heel were measured, and the former value was divided by the latter to calculate the arch index for each foot. | Not tested | Not tested | *r* = 0.45 | Nil | No | Protocol reported in cited article was Staheli arch index, protocol used in included study [53] was Chippaux-Smirak index |
| Clarke’s angle | [64] | NR | 3.7 – 11.7 | A static footprint was recorded for each foot during half body weight–bearing position. For each foot, the widest part of the arch and the heel were measured, and the former value was divided by the latter to calculate the arch index for each foot. | Not tested | Not tested | *r* = 0.45 | Nil | No | Protocol reported was for Staheli arch index, protocol used in included study [53] was Chippaux-Smirak index |
| Staheli arch index | [63] | NR | 25.0 (9.0) | Static and dynamic electronic footprint images obtained for each subject. The width of the forefoot and the heel were measured, the latter divided by the former and expressed as a percentage.  Images were then assessed for reliability of foot print indices measures between static and dynamic images. | Not tested | Not tested | Not tested | Nil | No | Adult population and protocol was comparing static to dynamic print indices |
|  | [65] | ≥0.26 | Mean age 21.5 (1.5) | Footprints obtained statically or dynamically with ink and paper. A line is drawn on the footprint from the center of the heel to the tip of the second toe (foot axis), second line is drawn perpendicular to the most anterior part of the main body (excluding toes). The foot is divided into equal thirds, dividing the foot into rearfoot (A), midfoot (B) and forefoot (C) regions. The total area of the footprint (A + B + C) and the arm in the midfoot (B) are then determined. Results on 107 adults were used to determine first and third quartiles – result is flatfoot as ≥ 0.26 | Not tested | Not tested | Not tested | Nil | No | Adult population and protocol reported was for Arch index, protocol used in included study [37] was Staheli arch index |
| Arch Height index | [69] | NR | Range 18 - 77 | Ratio of arch height to truncated foot length expressed as a percent whilst sitting with knee and ankle at 90 degrees | Not tested | Not tested | Not tested | Nil | No | Adult population and no validity testing conducted |
| FPI-6 | [72] | NR | Range 8 - 65 | Six items, (Talar head palpation, curves above and below lateral malleoli, inversion and eversion of the calcaneus, bulge in the region of the talonavicular joint, congruence of the medial longitudinal arch, and abduction and adduction of the forefoot on the rear foot (too-many-toes)) scored according to manual | Not tested | Not tested | Not tested | RASCH analysis. Good unidimensionality  (_12  2 test 11.49, *P*_.49),  & person-separation index of 0.88 | With caution | Includes adult population |

Study codes:

**57 – Gould 1982; 60 – McCrory et al. 1997; 63 – Mathieson, Upton & Birchenough 1999; 64 – Kanatli, Yetkin & Cila 2001; 65 – Cavanagh & Rodgers 1987; 69 – Hillstrom et al. 2013; 72 – Keenan et al. 2007.**

**Table A3:** Reported inter-rater reliability, population observed and QAREL score for included data

| **Measure** | **Study code or cited article** | **Definition of flat foot used** | **Age of population tested (Mean age ± (SD), range in years)** | **Inter-rater reliability**  **(ICC unless otherwise noted)** | **QAREL score** | **Was reliability reported for a paediatric population Yes/No/with caution** | **Cautions/Reasons for exclusion** |
| --- | --- | --- | --- | --- | --- | --- | --- |
| Arch Index | [43] | ≥ 0.26 | 10.4 (0.9), range 9 - 11 | LoA -0.02 to 0.01 | 3 | No | Intra-rater observation only |
| Chippaux-Smirak | [31] | ≥ 59% | 7.3 (1.1), range 6 – 9 | 0.98 | 3 | Yes |  |
|  | [43] | ≥ 45% | 10.4 (0.9), range 9 - 11 | LoA -1.18 to 2.91 | 3 | No | Intra-rater observation only |
| Clarke’s angle | [43] | ≤ 20° | 10.4 (0.9), range 9 - 11 | LoA -5.0 to 4.0 | 3 | No | Intra-rater observation only |
| Staheli arch index | [31] | ≥ 1.28 | 7.3 (1.1), range 6 – 9 | 0.95 | 3 | Yes |  |
| Martirosov’s K | [43] | ≥ 1.25 | 10.4 (0.9), range 9 - 11 | LoA -0.06 to 0.06 | 3 | No | Intra-rater observation only |
| Rearfoot eversion | [49] | > (7° - child’s age) | 5.1 (0.9), range 3 – 6 | 0.83 | 5 | Yes |  |
| Arch Height Index (sitting and standing) | [35] | ≤ 0.37 | 9.6 (2.0), range 6 – 13 | 0.76 to 0.89 | 5 | Yes |  |

LOA – Bland and Altman’s limits of agreement

Study codes:

**31 - Chang et al. 2014; 35 - Drefus et al. 2017; 43 - Nikolaidou & Boudolos 2006; 49 - Selby-Silversterin, Hillstrom & Palisano 2001.**

**Table A4:** Reported inter-rater reliability, population observed, protocol observed and QAREL score for cited data

| **Measure** | **Study code or cited article** | **Definition of flat foot used** | **Age of population tested (Mean age ± (SD), range in years)** | **Protocol applied** | **Inter-rater reliability**  **(ICC unless otherwise noted)** | **QAREL score** | **Was reliability reported for a paediatric population Yes/No/with caution** | **Cautions/Reasons for exclusion** |
| --- | --- | --- | --- | --- | --- | --- | --- | --- |
| Talus-first metatarsal | [58] | >4° | 47, range 20 – 57 | Patients were instructed to stand with the knee straight, their hands resting on a railing and the opposite foot non weight-bearing whilst lateral and anteroposterior (AP) plain film views were obtained. | r^2^ = 0.83 – 0.86 (lateral view) | 5 | No | Adult population |
| Arch Index | [61] | NR | 8.4 (1.7), range 5.5 – 11 | Footprint is marked with a “foot axis” line from the centre of the heel to the second toe. Two perpendicular lines to the foot axis are drawn at the most distant heel and forefoot areas (toes excluded). The foot axis line is separated into 3 equal sections defined as A (forefoot), B (midfoot), and C (rearfoot). The Arch Index is calculated as: B/A+B+C | r = 0.78 – 0.94 | 4 | Yes |  |
| Chippaux-Smirak | [62] | NR | 24.8 (2.1) | Two lines are drawn on foot print: one at the minimal distance of the midfoot region and one at the maximal distance of the forefoot area. The Chippaux-Smirak index is the minimal distance in the midfoot region divided by the maximal distance in the forefoot area | 0.96 | 3 | No | Adult population |
|  | [63] | NR | 25.0 (9.0) | Static and dynamic footprint images, three of each, were obtained for each subject using an electronic footprint system. The width of the forefoot and the heel were measured, the latter divided by the former and expressed as a percentage.  Images were then assessed for reliability of foot print indices measures between static and dynamic images. | Not tested | 2 | No | Adult population and assessing reliability between static and dynamic prints |
| Clarke’s angle | [63] | NR | 25.0 (9.0) | Static and dynamic footprint images, three of each, were obtained for each subject using an electronic footprint system. The Clarkes angle were measured as the angle between the medial reference line and a line connecting the most medial and anterior aspect of the medial longitudinal arch.  Images were then assessed for reliability of foot print indices measures between static and dynamic images. | Not tested | 2 | No | Adult population and assessing reliability between static and dynamic prints |
| Staheli arch index | [62] | NR | 24.8 (2.1) | Two lines drawn on footprint; one at the minimal distance of the midfoot region, and one at the maximal distance of the rearfoot region. The Staheli index is the ratio of the minimal distance in the midfoot region to the maximal distance in the rearfoot region. | 0.96 | 3 | No | Adult population |
| Footprint evaluation | [66] | NR | Range 3 – 17 | NR | Not tested | 1 | No | Article investigated Chippaux-Smirak and Clarke’s angle only. |
| Rearfoot eversion | [68] | NR | Range 6 – 16 | The examiner aligned the ankle with the foot at a right angle to the leg. The heel was aligned with the midline of the tibia with subtalar joint neither rotated in a varus or a valgus attitude. The Achilles tendon bisected with the straight edge of the goniometer, beginning proximally at the myotendinous junction and extending through the ankle joint down the posterior heel. | Not tested | 6 | No | Intra-reliability investigated only |
| Arch Height Index (sitting and standing) | [70] | NR | Range 18 – 45 | Subject either seated with knees flexed at 90°and feet resting on the floor or standing with 50% weight-bearing through each foot. One sliding caliper placed at end of the longest toe, one around the medial border of the first metatarsophalangeal joint. Truncated foot length obtained. A third caliper positioned at 50% foot length to determine dorsum height. Arch Height Index calculated as ratio of the height of the dorsum to the truncated foot length. | 0.99 | 3 | No | Adult population |
|  | [71] | NR | 29.9 (5.8) | Ratio of arch height to truncated foot length expressed as a percent whilst sitting with knee and ankle at 90 degrees. | Not tested | 5 | No | Adult population and intra-rater data supplied only |
| FPI-6 | [73] | NR | 10.6 (2.3), range 7 – 15 | The FPI-6 was evaluated as directed by the original protocol with each child standing in 50% weight-bearing per foot. | 0.79 | 5 | Yes |  |
|  | [74] | NR | Range 5 – 16 | Each participant was asked to stand, take a few steps forward and march on the spot for six-eight steps and then to stand still, with arms by their side and looking forward. Both observers performed an independent bilateral foot assessment of each child using the six criteria of the FPI-6:talar head palpation; curvature at the lateral malleoli; inversion/eversion of the calcaneus; talonavicular bulging; congruence of the medial longitudinal arch; abduction/adduction of the forefoot on the rearfoot | 0.86 (Kw) | 7 | Yes |  |

LOA – Bland and Altman’s limits of agreement

Study codes:

**58 – Younger, Sawatzky & Dryden 2005; 61 – Gilmour & Burns 2001; 62 – Queen et al. 2007; 63 – Mathieson, Upton & Birchenough 1999; 66 – Forriol & Pascual 1990; 68 – Sobel et al. 1999; 70 – Butler et al. 2008; 71 – Pohl & Farr 2010; 73 – Evans, Rome & Peet 2012; 74 – Morrison & Ferrari 2009.**

**Table A5:** QAREL checklist outcomes for inter-rater reliability data of included and cited articles.

| Study | Item Number | | | | | | | | | | |
| --- | --- | --- | --- | --- | --- | --- | --- | --- | --- | --- | --- |
|  | 1 | 2 | 3 | 4 | 5 | 6 | 7 | 8 | 9 | 10 | 11 |
| Butler et al. 2008 | Yes | Unclear | Unclear | Unclear | NA | Unclear | Unclear | Unclear | Unclear | Yes | Yes |
| Chang et al. 2014 | Yes | Unclear | Unclear | Unclear | Unclear | Unclear | Unclear | Unclear | Unclear | Yes | Yes |
| Drefus et al. 2017 | Yes | Yes | Unclear | Unclear | Unclear | Unclear | Unclear | Unclear | Yes | Yes | Yes |
| Evans, Rome and Peet 2012 | Yes | Yes | Yes | Unclear | Unclear | Unclear | Unclear | Unclear | Yes | Unclear | Yes |
| Forriol and Pascual 1990 | Yes | Unclear | Unclear | Unclear | Unclear | Unclear | Unclear | Unclear | Unclear | Unclear | Unclear |
| Gilmour & Burns 2001 | Yes | Yes | Unclear | Unclear | NA | Unclear | Unclear | Unclear | Unclear | Yes | Yes |
| Mathieson et al. 1999 | Yes | Unclear | No | Unclear | Unclear | Unclear | Unclear | Unclear | Unclear | Unclear | Yes |
| Morrison and Ferrari 2009 | Yes | Yes | Yes | NA | NA | Unclear | Unclear | Yes | Yes | Yes | Yes |
| Nikolaidou and Boudolos 2006 | Yes | Unclear | NA | Unclear | NA | Unclear | Unclear | Unclear | Unclear | Yes | Yes |
| Pohl and Farr 2010 | Yes | Yes | NA | Unclear | Unclear | Unclear | Unclear | Unclear | Yes | Yes | Yes |
| Queen et al. 2007 | No | Unclear | Unclear | Unclear | NA | Unclear | Unclear | Unclear | Yes | Yes | Yes |
| Selby-Silversterin, Hillstrom & Palisano 2001 | Yes | Unclear | Unclear | NA | NA | Yes | Yes | Unclear | Unclear | Yes | Yes |
| Sobel et al. 1999 | Yes | Yes | NA | Unclear | NA | Unclear | Unclear | Yes | Yes | Yes | Yes |
| Younger et al. 2005 | Yes | Unclear | Unclear | Unclear | NA | Yes | Yes | Unclear | Unclear | Yes | Yes |

References:

31. Chang C-H, Chen Y-C, Yang W-T, Ho P-C, Hwang A-W, Chen C-H, Chang J-H, Chang L-W: Flatfoot Diagnosis by a Unique Bimodal Distribution of Footprint Index in Children. PLoS One. 2014;9:e115808.

32. Chen KC, Yeh CJ, Kuo JF, Hsieh CL, Yang SF, Wang CH: Footprint analysis of flatfoot in preschool-aged children. Eur J Pediatr. 2011;170:611-617.

35. Drefus LC, Kedem P, Mangan SM, Scher DM, Hillstrom HJ: Reliability of the Arch Height Index as a Measure of Foot Structure in Children. Pediatr Phys Ther. 2017;29:83-88.

43. Nikolaidou ME, Boudolos KD: A footprint-based approach for the rational classification of foot types in young schoolchildren. Foot. 2006;16:82-90 89p.

45. Pauk J, Ihnatouski M, Najafi B: Assessing plantar pressure distribution in children with flatfoot arch: application of the Clarke angle. J Am Podiatr Med Assoc. 2014;104:622-632.

49. Selby-Silverstein L, Hillstrom H, Palisano R: The effect of foot orthoses on standing foot posture and gait of young children with Down Syndrome. Neurorehabilitation 2001;16:183-193.

53. Adoracion Villarroya M, Manuel Esquivel J, Tomas C, Buenafe A, Moreno L: Foot structure in overweight and obese children. Int J Pediatr Obes. 2008;3:39-45.

57. Gould N: Graphing the Adult Foot and Ankle. Foot & Ankle 1982, 2:213-219.

58. Younger AS, Sawatzky B, Dryden P: Radiographic assessment of adult flatfoot. Foot Ankle Int. 2005;26:820-825.

60. McCrory JL, Young MJ, Boulton AJM, Cavanagh PR: Arch index as a predictor of arch height. Foot. 1997;7:79-81.

61. Gilmour J, Burns Y: The measurement of the medial longitudinal arch in children. Foot Ankle Int. 2001;22:493-498.

62. Queen RM, Mall NA, Hardaker WM, Nunley JA, 2nd: Describing the medial longitudinal arch using footprint indices and a clinical grading system. Foot Ankle Int. 2007;28:456-462.

63. Mathieson I, Upton D, Birchenough A: Comparison of footprint parameters calculated from static and dynamic footprints. Foot. 1999;9:145-149.

64. Kanatli U, Yetkin H, Cila E: Footprint and radiographic analysis of the feet. J Pediatr Orthoped. 2001;21:225-228.

65. Cavanagh PR, Rodgers MM: The arch index: A useful measure from footprints. J Biomech. 1987;20:547-551.

66. Forriol F, Pascual J: Footprint analysis between three and seventeen years of age. Foot Ankle. 1990;11:101-104.

68. Sobel E, Levitz S, Caselli M, Brentnall Z, Tran MQ: Natural History of the Rearfoot Angle: Preliminary Values in 150 Children. Foot Ankle Int. 1999;20:119-125.

69. Hillstrom H, Song J, Kraszewski A, Hafer J, Mootanah R, Dudour A, Chow B: Foot type biomechanics part 1: Structure and function of the asymptomatic foot. Gait Posture. 2013;37:445-451.

70. Butler RJ, Hillstrom H, Song J, Richards CJ, Davis IS: Arch height index measurement system: Establishment of reliability and normative values. J Am Podiatr Med Assoc. 2008;98:102-106.

71. Pohl MB, Farr L: A comparison of foot arch measurement reliability using both digital photography and calliper methods. J Foot Ankle Res. 2010;3:14.

72. Keenan A, Redmond AC, Horton M, Conaghan PG, Tennant A: The Foot Posture Index: Rasch analysis of a novel, foot-specific outcome measure. Arch Phys Med Rehab. 2007;88:88-93 86p.

73. Evans AM, Rome K, Peet L: The foot posture index, ankle lunge test, Beighton scale and the lower limb assessment score in healthy children: a reliability study. J Foot Ankle Res. 2012;5:1.

74. Morrison SC, Ferrari J: Inter-rater reliability of the Foot Posture Index (FPI-6) in the assessment of the paediatric foot. J Foot Ankle Res. 2009;2:26.26.
