# Supplementary material for: Paediatric flexible flat foot: how are we measuring it and are we getting it right? A systematic review
Source: J Foot Ankle Res. 2018 May 30;11:21. doi: 10.1186/s13047-018-0264-3 (PMC5975578; doi:10.1186/s13047-018-0264-3)
Supplement: Supplementary file 2 — Table A4. Summary of foot posture tools. (DOCX 4276 kb) [file 13047_2018_264_MOESM2_ESM.docx]

**Additional file 2**

**Table A4:** Summary of foot posture tools

| Measure | Description |
| --- | --- |
| Calcaneal pitch | The angle between the plantar aspect of foot and line drawn along plantar surface of calcaneus most inferior projection (identified by red angle in Figure A1)    Figure A1: Calcaneal pitch |
| Anteroposterior talocalcaneal | The angle between the axis of the talus and calcaneus (identified by red angle in Figure A2).  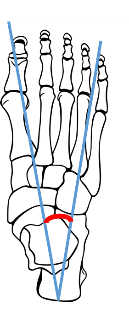  Figure A2: AP talocalcaneal |
| Plantarflexion (of talus) | No description offered. |
| Lateral talocalcaneal | The angle formed by the bisection of the talus and calcaneus (identified by red angle in Figure A3) [1].    Figure A3: Lateral talocalcaneal angle |
| Calcaneal first metatarsal | Tangent to the inferior surface of calcaneus and first metatarsal (identified by red angle in Figure A4)    Figure A4: Calcaneal first metatarsal angle |
| Talus-first metatarsal | Angle formed by intersection of talar and first metatarsal longitudinal axes (identified by red angle in Figure A5)    Figure A5: Talus-first metatarsal angle |
| Chippaux-Smirak index | Ratio of line B to line A where line A is at the maximum width at the metatarsals and line B is at the narrowest width of the midfoot (B/A x 100%).  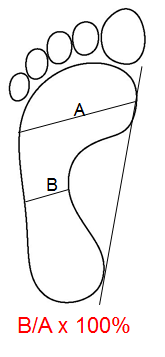  Figure A6: Chippaux-Smirak Index |
| Arch index | Foot axis is determined from centre of proximal heel and most anterior part in front of metatarsal heads, divided into equal thirds by parallel lines. Arch index is calculated as the ratio of the midfoot area to the area of the entire foot excluding toes (B/(A+B+C)).  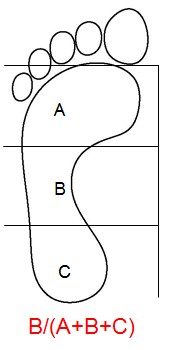  Figure A7: Arch index |
| Clarke’s angle | The angle between the most medial points of the metatarsal and heel areas and the apex of the concavity of the arch (identified by red angle in Figure A)  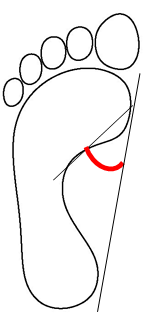  Figure A8: Clarke’s angle |
| Staheli arch index | The ratio of the narrowest point of the foot arch to the maximum width of the heel area (B/C x 100%)  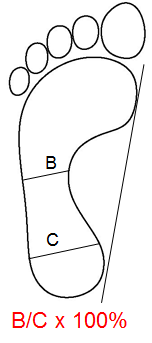  Figure A9: Staheli arch index |
| Martirosov’s K index | Horizontal lines bisecting foot are drawn at (A) most medial point of the metatarsal area, (B) most lateral point of the metatarsal area, (C) most lateral point of the heel area, (D) center of the heel area and from centre of the heel area to tip of the longest toe and first line from toe sulcus. Point E (point of intersection between line BC and a perpendicular line from point D to line BC) is marked and line BE is determined. Line BE is then divided into four parts equal to 0.16, 0.30, 0.46 and 0.60 of the length of the print. Points c, u, v and w located at the medial area of the footprint are marked and parallel lines, perpendicular to line BE, are drawn and marked as lines Cc, Uu, Vv, and Ww. Points F (middle of line Cc), G (located at the metatarsal area between the bases of the third and fourth toe) and Z (middle of line AB) are located and marked, and lines FG and FZ are drawn. Points H and K are the intersection points of lines FG, FZ and Vv. Lines x (width of the area enclosed between points H and the lateral outline of the footprint) and y (width of the area enclosed between points K and V) are determined and measured. KI is calculated as the ratio of line x to y.  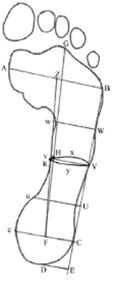  Figure A10: Martirosov’s K index [2] |
| Footprint evaluation | The calcaneal oval is identified, with the longitudinal axis of the foot (LAF) drawn from the center of calcaneus oval to the second toe and the longitudinal axis of calcaneus (LAC) bisecting the center of calcaneal oval. A medial tangential line is drawn for reference. A perpendicular line (y) is then drawn from the medial tangential line to the LAC, with a second line (x), drawn parallel to (y) joining the medial and LAF line. The result of y-x is the width of the isthmus of the footprint. Foot print classification is dependent on the y-x isthmus and classified into one of six foot print types (I, II, III, IV, V and VI) with line y reducing in length respectively across the types.  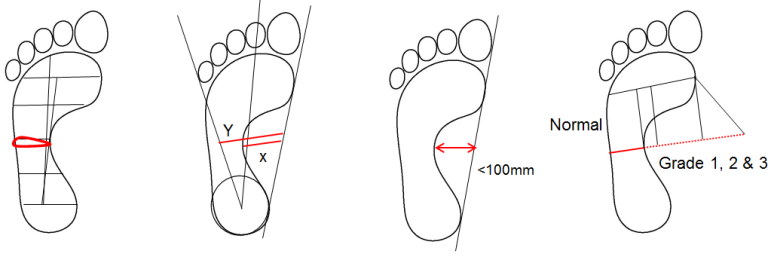  Figure A11: An example of a Type 1 Footprint evaluation. |
| Instep | Static weight bearing measure, assessing the footprint’s ‘instep’ value at its widest section (width <100 mm equals pes planus).  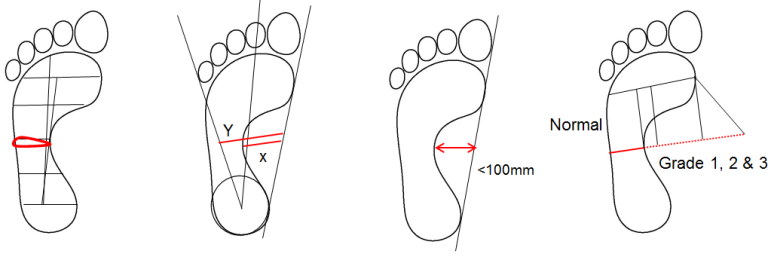  Figure A12: Instep |
| Plantar footprint | Flat foot is graded 1, 2 or 3. Grade 1 when the support of the lateral edge of the foot is half of that of the metatarsal support; grade 2 when the support of the central zone and forefoot are equal; and grade 3 in which the support in the central zone of the foot is greater than the width of the metatarsal support.  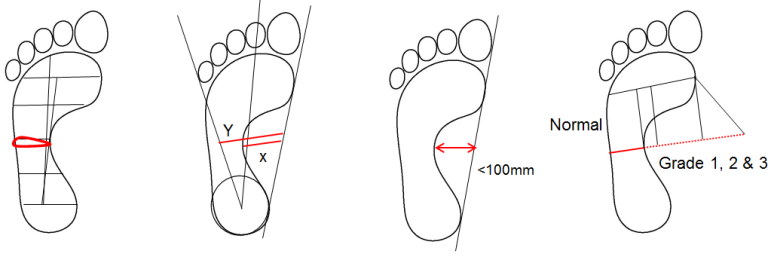  Figure A13: Plantar footprint |
| Rearfoot eversion | Angle between line through middle of posterior lower limb and calcaneal bisection. |
| Arch Height index | Ratio of arch height to truncated foot length expressed as a percent (whilst sitting standing or sitting). |
| FPI-6 | Six items scored a 0, 1 or 2 per item. 1). Palpation of talar head, 2). Curves above and below lateral malleoli, 3). Inversion and eversion of the calcaneus, 4). Bulge in the region of the talonavicular joint, 5). Congruence of the medial longitudinal arch, and 6). Abduction and adduction of the forefoot on the rear foot. |
| Navicular height | Not described in included study. Described elsewhere as the measurement, in mms, between the lowest point palpable of the navicular bone and the supporting surface [3]. |
| Foot Ground Pressure | Feet imprint captured by Footprint Measuring Instrument which records pressure (plantar pressure analysis) between foot and ground, by projecting circular inferences around load points. The diameter of each circle represents the magnitude of local load. |

**References**

1. Saltzman C, Nawoczenski D, Talbot K: Measurement of the medial longitudinal arch. Arch Phys Med Rehab. 1995;76:45 - 49.

2. Nikolaidou ME, Boudolos KD: A footprint-based approach for the rational classification of foot types in young schoolchildren. Foot. 2006;16:82-90.

3. Chang H-W, Lin C-J, Kuo L-C, Tsai M-J, Chieh H-F, Su F-C: Three-dimensional measurement of foot arch in preschool children. Biomed Eng Online. 2012;11:76-76.
